# *FOXP3* and *CTLA4* overexpression in multiple myeloma bone marrow as a sign of accumulation of CD4^+^ T regulatory cells

**DOI:** 10.1007/s00262-014-1589-9

**Published:** 2014-08-07

**Authors:** Walter Moises Tobias Braga, Bruna Raphaeli da Silva, Ana Carolina de Carvalho, Yumi H. Maekawa, Adriana Bruscato Bortoluzzo, Edgar Gil Rizzatti, Djordje Atanackovic, Gisele Wally Braga Colleoni

**Affiliations:** 1 Universidade Federal de São Paulo [UNIFESP], Rua Diogo de Faria, 824, 5º andar, Hemocentro, São Paulo, CEP 04037-003 Brazil; 2 Fleury Medicina e Saúde, São Paulo, Brazil; 3 Insper Institute of Education and Research, São Paulo, Brazil; 4 University Medical Center Hamburg-Eppendorf, Hamburg, Germany

**Keywords:** Multiple myeloma, FOXP3, CTLA4, Treg, Therapy

## Abstract

**Introduction:**

Multiple myeloma (MM) development involves a series of genetic abnormalities and changes in the bone marrow (BM) microenvironment, favoring the growth of the tumor and failure of local immune control. T regulatory (Treg) cells play an important role in dampening anti-tumor immune responses while T-helper-17 (Th17) cells seem to be critical for the eradication of malignant cells. The aim of our study was to characterize the expression of Treg- and Th17-related genes in total myeloma BM samples to assess their role as biomarkers, prognostic factors, and possible therapeutic targets in this incurable disease.

**Methods:**

Expression of markers for Treg (*FOXP3*, *CTLA4*) and Th17 cells (*RORγt*) was determined by quantitative real-time PCR in BM aspirates of 46 MM patients, four patients with monoclonal gammopathy of undetermined significance, five solitary plasmacytomas, and five healthy BM donors. Gene expression was evaluated regarding an influence on the patients’ overall survival (OS).

**Results:**

*FOXP3* and *CTLA4* presented a sixfold (*p* = 0.02) and 30-fold higher expression (*p* = 0.03), respectively, in MM patients than in controls. *RORγt* expression was similar in MM patients and controls. Median OS of MM patients was 16.8 (range 4.5–29.1) months, and international staging system was the only independent prognostic factor for patients survival.

**Conclusions:**

Overexpression of *FOXP3* and *CTLA4* in total BM samples suggests a local accumulation of immunosuppressive Tregs, the MM tumor environment, possibly dampening anti-tumor host immune responses. Therapeutic approaches targeting Treg cells and restoring local anti-tumor immunity may provide new treatment strategies for this incurable malignancy.

**Electronic supplementary material:**

The online version of this article (doi:10.1007/s00262-014-1589-9) contains supplementary material, which is available to authorized users.

## Introduction

Multiple myeloma (MM) development involves a series of genetic abnormalities and changes in the bone marrow (BM) microenvironment, favoring the growth of the tumor and the failure of local immune control. Cellular immune defects in MM include decreased number of CD19^+^ B cells as well as CD4^+^ and CD8^+^ T cells. The presence of these abnormalities seems to negatively correlate with survival, indicating a potential relationship between cellular immune components and disease control [1]. A significant impairment of T cell function has been described for MM patients and for patients with monoclonal gammopathy of undetermined significance (MGUS) [2].

Subpopulations of CD4^+^ T cells, such as T regulatory (Treg) and T-helper-17 (Th17) cells, have emerged as a key players in the immune control of cancer. Representing 5–10 % of all CD4^+^ T cells in the peripheral blood, Tregs express CD4 and CD25 surface antigens as well as cytoplasmic (cytotoxic T lymphocyte-associated antigen 4) CTLA4 and GITR. Treg cells control many facets of immune responses in an attempt to maintain immune homeostasis, ranging from autoimmune diseases to inflammatory conditions and cancer. Two major Treg populations have been reported to date. Natural Treg (nTreg) cells develop in the thymus and constitute a critical arm of active mechanisms of peripheral tolerance, particularly to self-antigens. A growing body of knowledge supports the existence of induced Treg (iTreg) cells, which may derive from conventional CD4+ T cells. CD4+ T cells are induced by IL-10 and secrete both IL-10 and transforming growth factor β (TGFβ) converting peripheral naïve CD4+ CD25− FOXP3− cells to FOXP3-expressing cells. Regardless of their origin, they share one key feature: their ability to potently suppress effector T cells (Teff). This suppressive activity of Treg cells is associated with the overexpression of FOXP3, a member of the forkhead/winged helix family, which acts as a transcriptional repressor [3], and CTLA4, a coinhibitory molecule that maintains immune homeostasis by down regulating T cell-related costimulatory pathways. FOXP3-expressing cells in cancer settings have been correlated with poor anti-tumor effector response, hence compromising tumor immunity [4, 5].

Currently, many studies are focusing on Treg populations in MM, and a lot of controversy has been found in this field. While Prabhala et al. [2] showed an increase in CD4+ CD25+ in peripheral blood and BM of monoclonal gammopathy of undetermined significance (MGUS) and MM patients when compared with controls, they also found that Treg cells—evaluated by FOXP3 expression—are significantly decreased in number and function in patients with MGUS and MM compared with healthy donors. In other study, Gupta et al. [6] reported a decrease in frequency of Treg as well as reduced expression of FOXP3 by flow cytometry in peripheral blood samples of untreated MM patients, which increased significantly after treatment with thalidomide. Furthermore, Treg MM cells exhibited immunosuppressive activity in vitro [6]. On the other hand, Beyer et al. [7] demonstrated in both MGUS and MM that functional FOXP3+ Treg cells of naive, central, and effector memory phenotype (evaluated by CCR7 and CD45RA expression) are significantly expanded. The last study was supported by Feyler et al. [8] that showed significantly elevated levels of functional CD4+ CD25+ FOXP3+ Treg cells obtained from peripheral blood samples and analyzed by flow cytometry in MGUS and MM in comparison with healthy controls.

Th17 cells, a recently described IL-17-expressing CD4^+^ T cell subset, protect hosts against fungal and parasitic infections and participate in inflammatory reactions and autoimmunity. One of the main Th17-specific transcription factors is the orphan nuclear receptor, RORγ. Its specific isoform RORγt is selectively expressed by Th17 cells and regulated by STAT3. Overexpression of *RORγt* promotes Th17 differentiation while inhibiting Th1 and Th2 development [9, 10]. The role of Th17 cells in tumor pathogenesis is still not well defined. However, it seems that the balance between Treg and Th17 cells is particularly important for maintaining the homeostasis of anti-tumor immunity [11]. FOXP3 plays a negative role in IL-17 expression through physical interaction with RUNX1 and RORγt, thereby inhibiting their transactivation activity. RUNX1 is highly expressed in CD4^+^ T cells as well as FOXP3^+^ Tregs and interaction between RUNX1 and FOXP3 is essential for FOXP3 function. RUNX1 also has a significant role in Th17 differentiation due to its ability to induce RORγt expression and to associate with and to act together with RORγt to induce *IL17* transcription [12].

Increased number of Th17 cells in MM BM in comparison with peripheral blood has also been described. Dhodapkar et al. [13] demonstrated that the proportion of Th17 cells in the BM of MM patients was higher than in peripheral blood samples. Moreover, functional properties varied according to compartment: MM BM samples contained a significantly higher proportion of polyfunctional Th17-1 cells than peripheral blood, and these cells were not observed in MGUS BM samples. IL-6, TGFβ, and IL-1 play an important role in MM immune dysfunction. Since IL-6 and TGFβ are also important for the generation of Th17 cells, Prabhala et al. [11] observed a significantly elevated baseline and induced frequency of Th17 cells in peripheral blood and BM from MM patients compared with controls. They confirmed a significant increase in IL-17, IL-21, IL-22, and IL-23 serum levels in blood and BM in MM compared with healthy controls and that IL-17 promotes MM cell growth and colony formation via IL-17 receptor, adhesion to BM stromal cells and increased growth in the in vivo murine xenotransplant model of human MM cell lines. The authors concluded that Th17 cells play an important role in MM pathobiology and may be an important therapeutic target for anti-tumor effect [11].

In this scenario, we believe that the balance between Treg and Th17 cells is important in the BM of MM. Also, exploring this concept may be more relevant than previous results that used exclusively peripheral blood samples or analyses only one of these CD4^+^ populations. Therefore, the aim of this study was to simultaneously characterize the expression of Treg (*FOXP3* and *CTLA4*)- and Th17 (*RORγt*)-related genes in total MM BM samples in order to assess the local immune milieu as potential biomarker/therapeutic target and to understand whether these genes have a prognostic impact on this incurable disease.

## Materials and methods

### Patients

Forty-six newly diagnosed MM patients referred to the Hematology and Hemotherapy Service of Federal University of São Paulo (UNIFESP) in São Paulo, Brazil were collected between 2003 and 2012. Only patients with no previous chemotherapy, corticosteroid, or bisphosphonate treatment were included. The diagnosis of MM was based on The International Working Group Criteria [14], and information on tumor stage was obtained for all patients according to Durie–Salmon and the international staging system (ISS) [15]. According to the ISS, 6 cases were classified as stage I, 13 as stage II, 23 as stage III, and 4 cases could not be classified because of the absence of beta2-microglobulin data (Table 1). Patients were treated with dexamethasone/thalidomide or melphalan/prednisone/thalidomide, depending on their age and performance status, and 5 patients also received high-dose melphalan followed by autologous stem cell transplantation. We also studied BM aspirates from 4 patients with MGUS, 5 patients with solitary plasmacytoma (SP), and 5 healthy BM donors (not age matched with patients’ group). Written informed consent was obtained from all patients and controls, and the study was approved by the Ethical Committee of this institution.

**Table 1:** Multiple myeloma characteristics at diagnosis (*N* = 46)

Characteristics	
Median age, years (range)	66 (27–95)
*Sex, n (%)*	
Male	25 (54)
Female	21 (46)
*Type of M-protein, n (%)*	
IgA	09 (20)
IgG	25 (54)
IgM	01 (02)
Light chain disease	04 (09)
NA	07 (15)
*D&S stage, n (%)*	
I	02 (04)
II	01 (02)
III	43 (94)
*ISS*	
1	06 (13)
2	13 (28)
3	23 (50)
NA	04 (09)

*D&S* Durie–Salmon classification, *ISS* international staging system, *NA* not available

### Magnetic sorting of CD138-positive cells

BM samples were collected in sterile EDTA tubes during routine diagnostics, and neoplastic plasma cells were separated by magnetic sorting of CD138-positive cells using the MACS system (Magnetic Cell Sorting of Human Cells; Miltenyi Biotec, Bergisch-Gladbach, Germany), according to previous descriptions [16].

### Flow cytometry

For the analysis of Treg cells in total BM, the flow through (after CD138^+^ sorting) cells of 13 MM, 1 SP, 2 MGUS patients, and 3 healthy donors were stained with allophycocyanin (APC)-H7-Clone SK7-labeled anti-CD3, peridinin–chlorophyll–protein complex (PerCP)-Clone SK3-labeled anti-CD4 and fluorescein isothiocyanate (FITC)-M-Clone A251-labeled anti-CD25 antibodies (BD Biosciences, NSW, Australia). Intracellular labeling with PE-conjugated anti-FOXP3-Clone 236a/E7 and anti-CTLA4-APC-Clone BNI3 was followed by permeabilization with fix/perm solution according to the manufacturer’s protocol (BD Biosciences). Flow cytometry was carried out using a BD FACScanto II (BD Biosciences), and lymphocytes were gated based on their forward and side light scatter properties. Data were analyzed with Infinicyt software (Cytognos SL, Salamanca, Spain). After gating the lymphocyte population, CD3^+^CD4^+^CD25^high^FOXP3^+^CTLA4^+^ populations showing a Treg phenotype were sequentially gated and analyzed [17, 18]. Proportional numbers of Treg cells were calculated from target cell frequency as determined by flow cytometry and total lymphocyte counts calculated during routine diagnostics.

### RNA extraction

RNA extraction from mononuclear cells (MNC) derived from total BM was performed as previously described [19]. Expression of genes related to the Treg and Th17 subpopulations, respectively, was evaluated by quantitative real-time PCR (qPCR) using 7500 Real Time PCR System (Applied Biosystems, Foster City, CA). Commercially available qPCR assays were used: *FOXP3* (Hs01085834_m1*), *CD25* (Hs00907779_m1*), *TGFβ1* (Hs00998133_m1*), *CTLA4* (Hs03044418_m1*), *RORyt* (Hs01076112_m1*)*, IL6* (Hs00985639_m1*), and *IL*-*17A* (Hs00174383 m1*).

Expression of target genes was normalized by the arithmetic mean of triplicates of housekeeping genes (*ACTβ* and *GAPDH*) [19]. Samples containing no cDNA were used as negative controls. All samples were analyzed in triplicate. The relative mRNA expression level of the target genes was calculated using the 2^−ΔΔCT^ method [20]. Genes were considered differentially expressed in tumor samples when their expression levels showed at least a twofold increment or decrease in comparison with normal samples.

### Statistical analysis

Associations between the variables were tested by the Pearson’s chi-squared test (*χ* ^2^). The Mann–Whitney test was used to compare two individual values. Pearson’s product-moment correlation coefficient (Pearson’s *r*) was used to measure the strength of linear dependence between two variables. Overall survival (OS) was calculated from the date of diagnosis of MM until death or last follow-up. Actuarial probabilities of OS were estimated according to the Kaplan–Meier method, and the curves were compared using the log-rank test. Cox’s regression model was also employed to evaluate which variables could be considered independent prognostic factors. Differences with a *p* < 0.05 were regarded as statistically significant. Statistical analyses were performed using SPSS Software version 14.0 (SPSS Inc., Chicago, IL) and Prism 5 software (GraphPad, San Diego, CA).

## Results

**Fig. 1:**
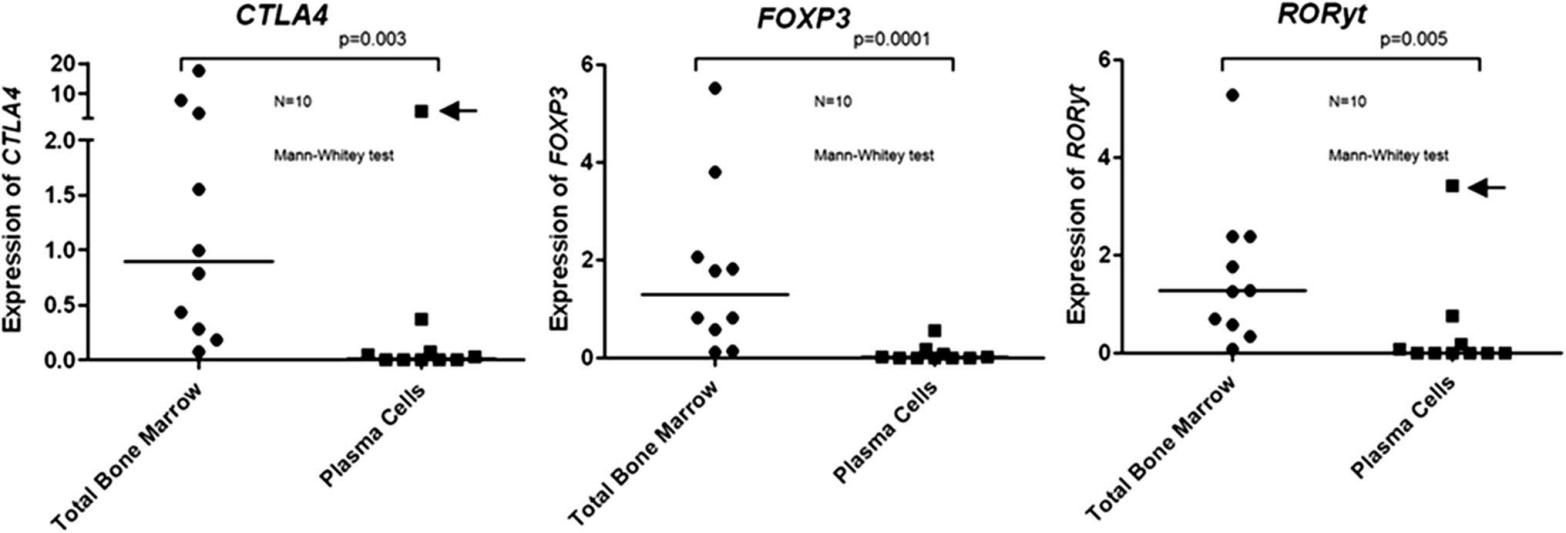
Relative expression (2^−ΔΔCT^) of *FOXP3, CTLA4,* and *RORyt* in sorted plasma cells and their respective total bone marrow aspirates by qPCR. Pilot study with 10 MM cases shows that Treg and Th17 genes were not significantly expressed by plasma cells, with the exception of one outlier (*arrows*) for *CTLA4 and RORyt*

The present study aimed at describing the bone marrow environment of MM with regard to the presence of Tregs and Th17 cells. Determining the frequency of these immunomodulating T cell subtypes in whole BM could theoretically be hampered by expression of the respective marker genes by tumor cells themselves. Therefore, in a first step, we checked whether *FOXP3, CTLA4,* and *RORyt* were aberrantly expressed by malignant plasma cells. The results showed a 125-fold lower *FOXP3* expression (*p* = 0.0001), 52-fold lower *CTLA4* expression (*p* = 0.003), and almost undetectable *RORyt* expression (*p* = 0.005) when plasma cells samples were compared to total BM (Fig. 1). These data are in agreement with Oncomine cancer profiling database (www.oncomine.org). Therefore, we concluded that the genes analyzed in this study are indeed not expressed by BM-infiltrating myeloma cells, and their expression in whole BM is mainly determined by the local presence of Tregs and Th17 cells.

**Fig. 2:**
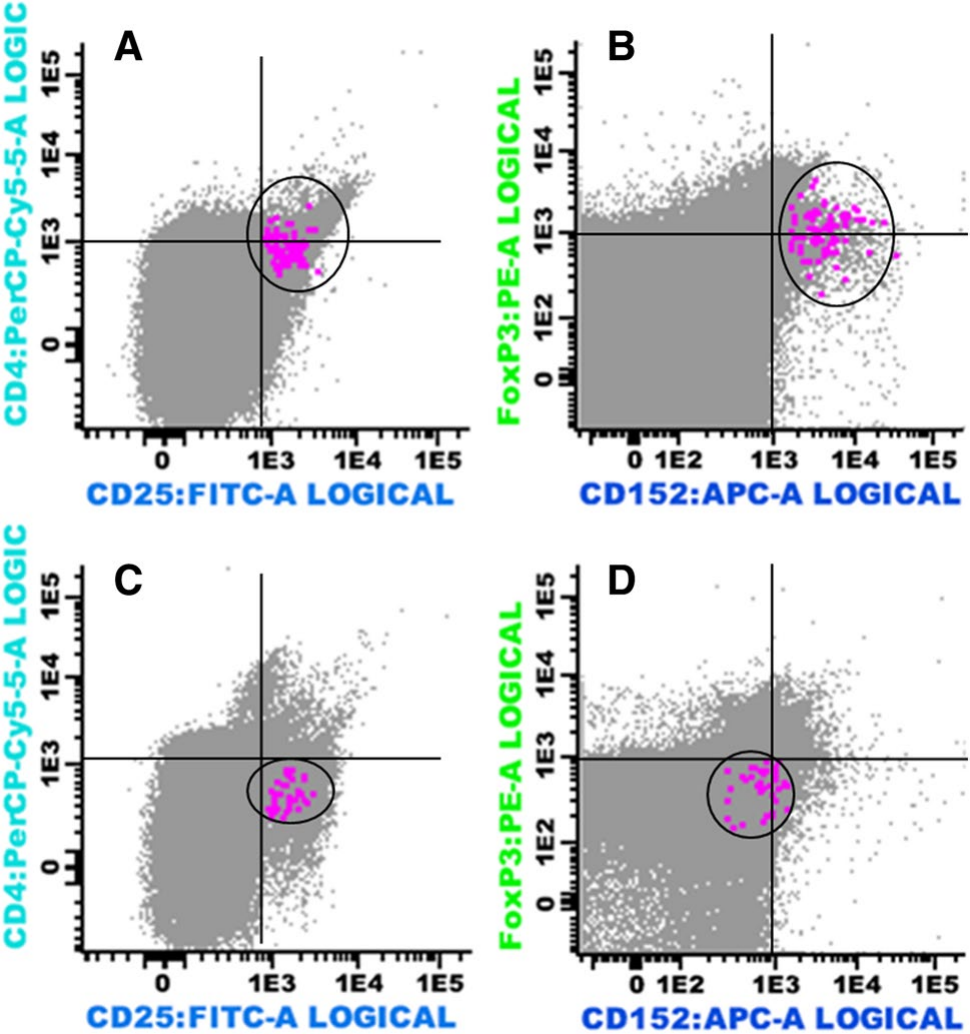
Multiparameter flow cytometry analysis showing CD4^+^ CD25^high^ immunophenotype in bone marrow aspirate samples from a case of multiple myeloma (**a**) and a healthy donor (**c**) (indicated as *color dots*); FOXP3^+^CTLA4(CD152) positive (*color dots*) in the same myeloma sample **(b**) and negative in the same healthy donor sample (**D**)

**Fig. 3:**
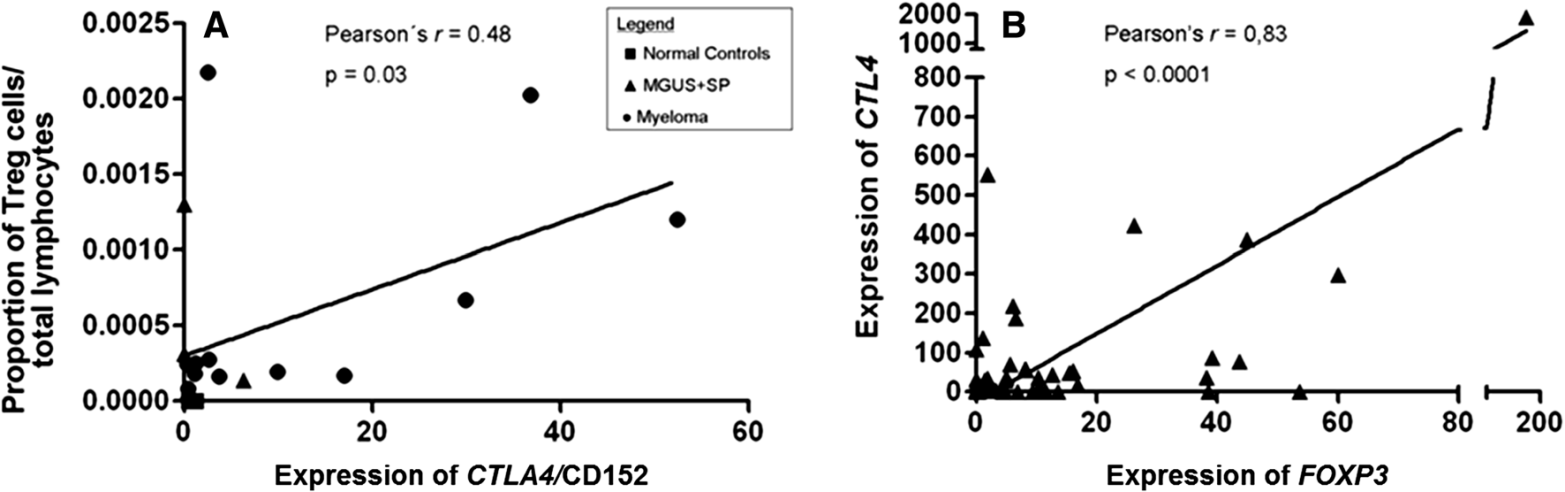
**a** Linear correlation between the proportion of Treg cells/total lymphocytes in BM samples by flow cytometry and *CTLA4* (CD 152) expression by qPCR in 19 samples: 13 cases of MM, 1 SP, 2 MGUS patients, and 3 normal controls. **b** Linear correlation between expression of *FOXP3* and *CTLA4* in 46 bone marrow aspirates of multiple myeloma (MM) by qPCR

Next, we analyzed whether real-time PCR would be as useful for quantification of immunomodulating T cell subsets as flow cytometry (considered the gold standard method in most of the previous studies). Applying both methods to 19 BM samples (13 cases of MM, 1 SP, and 2 MGUS patients), as well as 3 healthy donors, we found a linear correlation (*r* = 0.48; *p* = 0.03) between the proportion of Tregs (CD3^+^CD4^+^CD25^high^FOXP3^+^CTLA4^+^) in total BM samples as determined by flow cytometry and the expression of *CTLA4* assessed by qPCR (Figs. 2, 3a and Supplementary Figure 1).

**Fig. 4:**
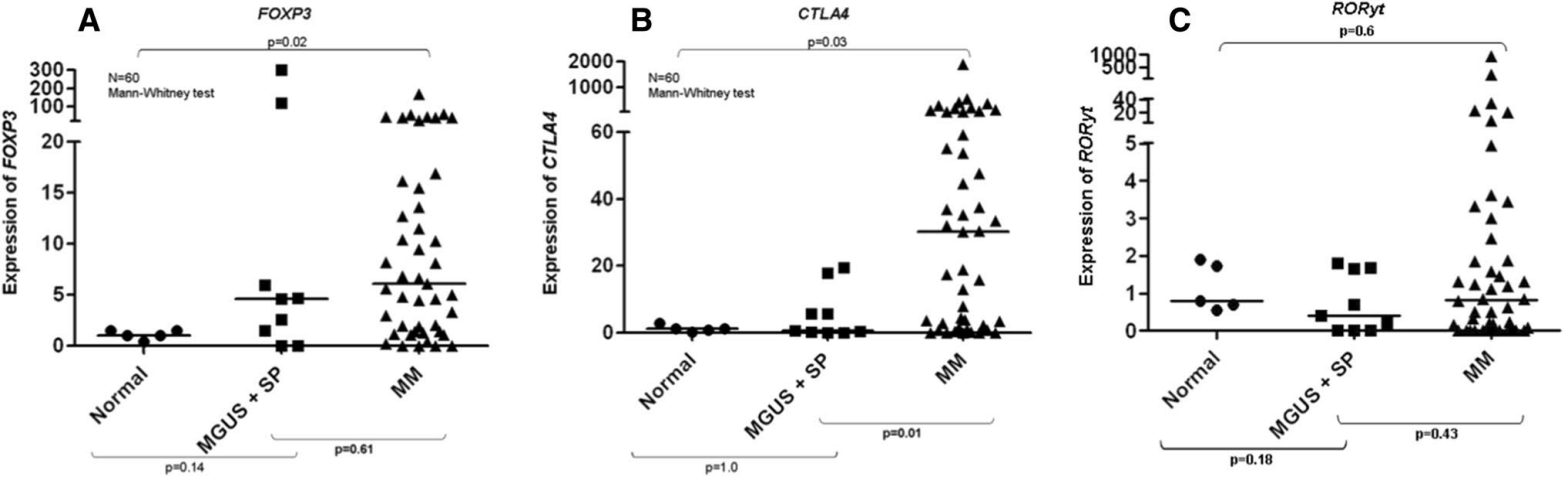
**a** Relative expression (2^−ΔΔCT^) of *FOXP3* in bone marrow aspirates of multiple myeloma (MM), solitary plasmacytomas (SP), monoclonal gammopathy of undetermined significance (MGUS) patients and normal controls by qPCR. **b** Relative expression (2^−ΔΔCT^) of *CTLA4* in bone marrow aspirates of multiple myeloma (MM), solitary plasmacytomas (SP), monoclonal gammopathy of undetermined significance (MGUS) patients and normal controls by qPCR. **c** Relative expression (2^−ΔΔCT^) of *RORyt* in bone marrow aspirates of multiple myeloma (MM), solitary plasmacytoma (SP), monoclonal gammopathy of undetermined significance (MGUS) patients and normal controls by qPCR

Having shown that real-time PCR is useful to quantify Tregs and Th17 cells in whole BM samples from myeloma patients, we applied this approach to all samples collected. Treg-related gene *FOXP3* was overexpressed in 68 % of individual MM cases, and overall expression was 6.1-fold higher (*p* = 0.02) in MM patients when compared to healthy donors (Fig. 4a). *CTLA4* was overexpressed in 76 % of individual MM cases, and overall expression was 30-fold higher when compared to controls (*p* = 0.03) (Fig. 4b). A strong linear correlation was observed between the expression of genes *FOXP3* and *CTLA4* (Pearson’s’ *r* = 0.83 and *p* < 0.0001) (Fig. 3b). In contrast, expression of *RORγt* was not different when MM patients and healthy controls were compared (Fig. 4c). We also analyzed BM expression of other genes related to Treg and Th17 cell subpopulations. However, BM expression levels of *CD25, IL6,* and *TGFβ1* were equivalent in MM patients and healthy controls. IL-17 showed no expression in normal or MM BM aspirates using two different primer sequences. Median OS of MM patients was 16.8 months (range 4.5–29.1). Univariate analysis indicated that none of the CD4^+^ T cell-related genes but only ISS (*p* = 0.011) had impact on the patients’ prognosis, a finding confirmed by Cox regression analysis (*p* = 0.011, RR 5.04, CI 1.15–22.11) (Table 2).

**Table 2:** Univariate analysis: overall survival according to ISS, *FOXP3*, *CTLA4,* and *RORyt* expression

Factor	*N* (%)	Median OS (months)	*p* value
*ISS*			0.011
1	06 (13)	36.3	
2	13 (28)	42.3	
3	23 (50)	5.4	
*FOXP3*			0.690
Underexpressed	07 (15)	3.0	
Normal expression	08 (17)	29.0	
Overexpressed	31 (67)	16.8	
*CTLA4*			0.817
Underexpressed	06 (13)	36.3	
Normal expression	05 (11)	19.9	
Overexpressed	35 (76)	11.6	
*RORyt*			0.447
Underexpressed	21 (46)	16.8	
Normal expression	13 (28)	29	
Overexpressed	12 (26)	2.5	

*NA* not available

## Discussion

In this study, we observed the overexpression of *FOXP3* and *CTLA4* in the BM of myeloma patients when compared to healthy controls. In contrast, Th17-related genes were not differentially expressed in the BM of patients with MM. The virtual absence of *FOXP3* and *CTLA4* expression in myeloma cells and the linear correlation of *CTLA4* gene expression with that of BM-infiltrating Tregs by flow cytometry led us to believe that most of the expression of Treg-related genes in myeloma BM aspirates might be due to the presence of the immunosuppressive CD4^+^ T cell subpopulation in tumor microenvironment.

Therefore, our finding of an increased expression of *FOXP3* and *CTLA4* in BM of MM patients suggests an accumulation of immunosuppressive Tregs in the tumor microenvironment of myeloma patients. We believe that local accumulation of Tregs in the lack of major changes in Th17 gene expression could contribute to the immune imbalance in MM. Of note, accumulating evidence indicates that Th17 cells may provide protection against malignancies by inducing Th1-type chemokines and recruiting effector cells to the tumor site [21]. Tregs, on the other hand, are believed to maintain immunologic tolerance to tumor cells by suppressing the activation and expansion of self-reactive immune cells [22, 23].

We thought to use total BM as the source of samples for this study since our institution has RNA bank available and the study of the T cell compartment inside tumor BM environment, without manipulation of CD4 subpopulations by sorting looked appealing. A pilot study using ten sorted plasma cells samples (and their respective total BM) supported the hypothesis that MM tumor cells are not responsible for Treg- or Th17-related genes expression. The small amount of *FOXP3, CTLA4, or RORyt* detected (including one outlier for the last two genes) in plasma cells samples could be related to “contamination” of sorted cells with BM T cells.

Despite FOXP3 being considered the most specific marker for naturally occurring CD4^+^ CD25^+^ Tregs, dividing activated T effector cells (Teff cells) can express FOXP3 transiently, and at significantly lower levels than suppressive Tregs [24]. Therefore, in this study, *FOXP3* transcripts were attributed, not exclusively but mostly, to Tregs in total BM.

To validate further gene expression studies, we analyzed 19 samples by both qPCR and flow cytometry, in which the flow through (after CD138^+^ sorting) was also available. While there is a linear correlation between the proportion of Treg cells/total lymphocytes in BM samples by flow cytometry and *CTLA4* expression by qPCR in 19 samples, it is also clear that for three of the 16 samples (one MM and 2 MGUS/PS) qPCR and flow cytometry data do not correlate. One possible explanation lies on gene expression regulation both at the transcriptional level and at the posttranscriptional level by posttranslational modifications.

There are a number of studies that have used FOXP3 qPCR as a surrogate for Tregs, with or without previous CD4-positive population sorting [25–27] or ROR*yt* as a surrogate marker of Th17 population [13]. Liu et al. [25] used a similar approach to compare qPCR and flow cytometry adopted in Fig. 3a, to correlate the percentage of demethylation of FOXP3 by real-time PCR and the one of CD4^+^ CD25^hi^ T cells by flow cytometry in cord blood mononuclear cells.

It is important to consider that the obtaining of an “ideal” bone marrow healthy donor is a challenge in this type of study, where the median age of MM patients was 66 years old, ranging from 27 to 95 years. Therefore, in the absence of a better alternative, we used healthy controls that were not age matched, recruited after informed consent among bone marrow donors for allogeneic transplant. We can see in Fig. 4b that there is a significant difference in *CTLA4* expression between MGUS/PS and MM independent of the age of controls.

The majority of patients in our study was diagnosed with advanced disease (stage III of Durie & Salmon: 94 % and ISS 2*/*3: 78 %) and, despite a significant increase in the expression of genes related to Treg cells, this had no impact on MM OS. Unfortunately, advanced stage MM is part of the reality of all public hospitals in Brazil. In a multicenter study, Hungria et al. [28] found 76.5 % of Durie and Salmon stage III MM cases and 79.9 % ISS 2/3 among 1066 analyzed patients.

In the survival analysis illustrated in Table 2, the markers are scored in three categories, using the same cutoffs defined for gene expression analyses (twofold increment or decrease in comparison with normal samples). Unfortunately, the small number of samples analyzed, and the treatment heterogeneity (only five patients were eligible for autologous stem cell transplantation) also could reduce the chances of achieving a correlation between the expression of the studied genes and clinical outcome.

However, recent studies addressed this topic using different methods and could demonstrate that the percentage of Treg cells isolated from peripheral blood and or bone marrow could have impact in MM outcome. In Giannopoulos et al. [23] study, patients were divided into two cohorts according to median Treg frequency. Those with a high percentage of Treg lived significantly less (median overall survival [OS] of 21 months) as compared with those with lower Treg frequency (median OS not reached at median follow-up of 32 months). The difference in survival was also observed when only non-transplanted patients were analyzed. On the other hand, patients submitted to autologous transplant presented no difference in OS when both groups of Treg frequencies were compared [29]. Raja et al. [30] evaluated Treg cells in both peripheral blood and bone marrow in a larger number of cases of MM using flow cytometry. They found a significantly elevated frequency of Treg cells in a newly diagnosed and relapsed MM patients compared with healthy controls. Functional studies showed that Treg cells from both MM and controls were similar in their inhibitory function. Using cutoffs very close to Giannopoulos et al. group [23], they showed that MM patients with high percentage of Treg cells had inferior time to progression and this variable had independent prognostic impact by multivariate analysis [30].Unfortunately, our study could not confirm the above results using two Treg-related gene expressions.

In this study, we also analyzed BM expression of other genes related to Treg and Th17 cell subpopulations. However, BM expression levels of *CD25* and *TGFβ1* (Treg-related genes) were equivalent in MM patients and healthy controls, and IL-17 (Th17-related gene) showed no expression in normal or MM bone marrow aspirates using two different primer sequences. The results were positive only after PCR product reamplification by qPCR. To avoid discrepancies with the expression of other genes, we decided to use only *RORyt* as a surrogate (and more reliable) marker for Th17. However, it failed to detect any abnormality when normal samples were compared to MM BM aspirates.

An imbalance between Treg and Th17 cells comparable to the one we have observed in the BM of our MM patients has been previously demonstrated in the tumor microenvironment of other malignancies. For example, one study in gastric cancer showed an increased number of Th17 cells as well as Treg in the tumor microenvironment in early stages of the disease. With progression of the cancer, tumor infiltration by Th17 cells gradually decreased while numbers of Tregs increased [31]. It is important to emphasize that Treg and Th17 developmental programs are reciprocally interconnected: upon T cell receptor (TCR*)* stimulation, naive T cells can be driven to express FOXP3 and become Tregs, if TGFβ is present. However, in the presence of TGFβ plus IL-6 or IL-21, the developmental pathway of Tregs is abrogated, and instead, T cells develop into Th17 cells. Only the combination of TGFβ plus IL-6 and IL-21, but neither one of them alone, induces a robust production of IL-17 by naive T cells [32, 33]. Therefore, IL-6 plays a pivotal role in dictating the balance between the generation of Tregs and Th17 cells as indicated by the *FOXP3/RORγt* ratio [5, 34]. Based on the well-known fact that in myeloma, IL-6 is produced in an autocrine and paracrine fashion and promotes the survival and progression of tumor cells; we had expected that the induction of Th17 would have been favored in this malignancy [35]. However, it seems that in MM other factors are involved instead to promote the local accumulation of Tregs and not the development of Th17 cells.

In the opposite direction of our findings, there is also an interesting study in a humanized murine model, showing that ex vivo-cultured human Tregs do not suppress anti-tumor immunity in BM, but only if the tumor is located outside the BM [34]. Again, using the Treg/Th17 reciprocally interconnected theory, the authors demonstrated that BM stromal cells reverse the suppressive activity of Tregs and promote IL-17 expression in these cells via IL-1β and IL-6 production. Therefore, the authors feel skeptical about the benefits of future Treg-based therapies [36]. Also, BM stromal cell may have an important role in maintaining Treg/Th17 interconnection in MM.

In conclusion, our current study suggests an accumulation of immunosuppressive Tregs in tumor microenvironment of myeloma patients by an overexpression of *FOXP3* and *CTLA4*. Even in a still controversial scenario, we believe that this finding could bring new insights about possible biomarkers and therapeutic targets for MM. Monoclonal antibodies targeting CTLA4, such as ipilimumab, should be evaluated in myeloma as they have been, for example, in melanoma [5]. Alternatively, the depletion of Treg cells, with a possible “reprogramming” of these cells to pro-inflammatory cells, could be a strategy of immunotherapy against human malignancies such as MM [37].

### Electronic supplementary material

Below is the link to the electronic supplementary material.

Supplementary material 1 (PDF 305 kb)

## Abbreviations

BM: Bone marrow
CTLA4: Cytotoxic T lymphocyte-associated antigen 4
FOXP3: Member of the forkhead/winged helix family
IL: Interleukin
iTreg: Induced Treg
ISS: International staging system
MGUS: Monoclonal gammopathy of undetermined significance
MM: Multiple myeloma
nTreg: Natural Treg
OS: Overall survival
qPCR: Quantitative real-time PCR
RUNX1: Runt-related transcription factor 1
RORγt: Orphan nuclear receptor γt
SP: Solitary plasmacytoma
STAT 3: Signal transducer and activator of transcription 3
TCR: T cell receptor
Teff: T effector cells
TGFβ: Transforming growth factor β
Th1: T-helper 1
Th2: T-helper 2
Treg: T regulatory
Th17: T-helper-17
