# Supplementary material for: FOXP3 and CTLA4 overexpression in multiple myeloma bone marrow as a sign of accumulation of CD4+ T regulatory cells
Source: Cancer Immunol Immunother. 2014 Aug 7;63(11):1189–97. doi: 10.1007/s00262-014-1589-9 (PMC4209089; doi:10.1007/s00262-014-1589-9)
Supplement: Supplementary file 1 — Supplementary material 1 (PDF 305 kb) [file 262_2014_1589_MOESM1_ESM.pdf]

Supplementary Figure 1

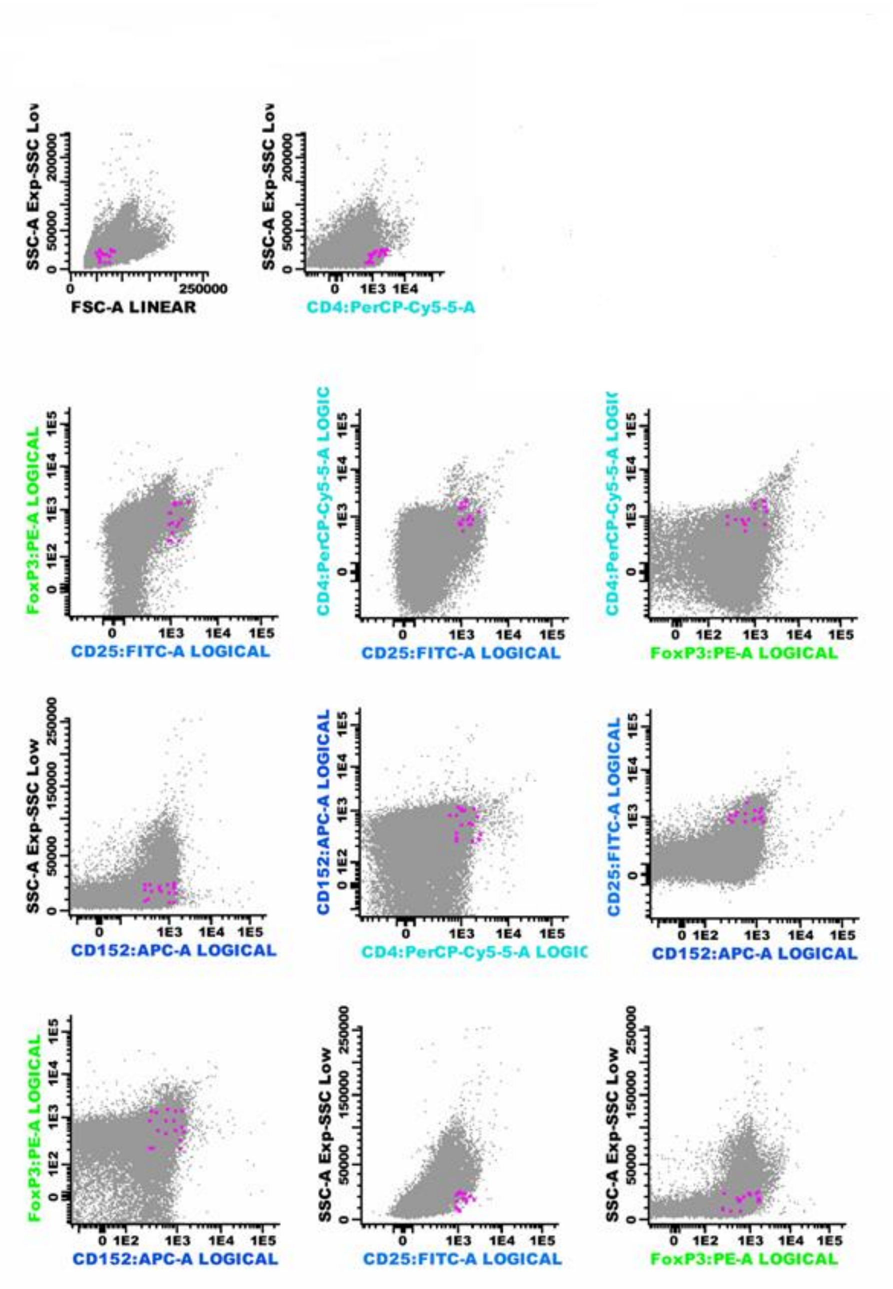

Supplementary Figure 1 A - Analysis of Treg cells by flow cytometry in total bone marrow aspirate: CD3+CD4+CD25highFOXP3+CTLA4(CD152)+ cells in a solitary plasmacytoma case.

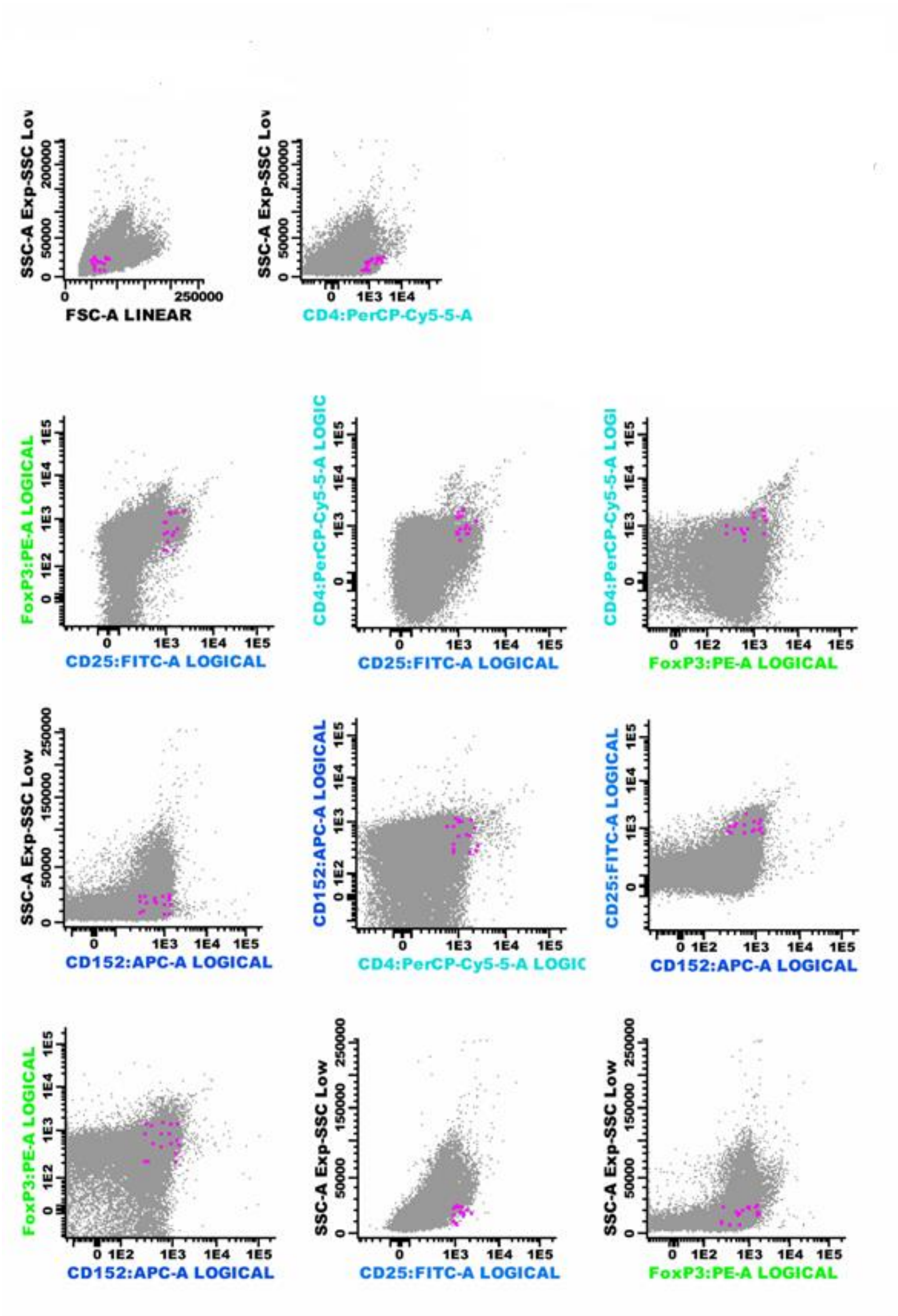

Supplementary Figure 1 B - Analysis of Treg cells by flow cytometry in total bone marrow aspirate: CD3+CD4+CD25highFOXP3+CTLA4(CD152)+ cells in a healthy donor sample.

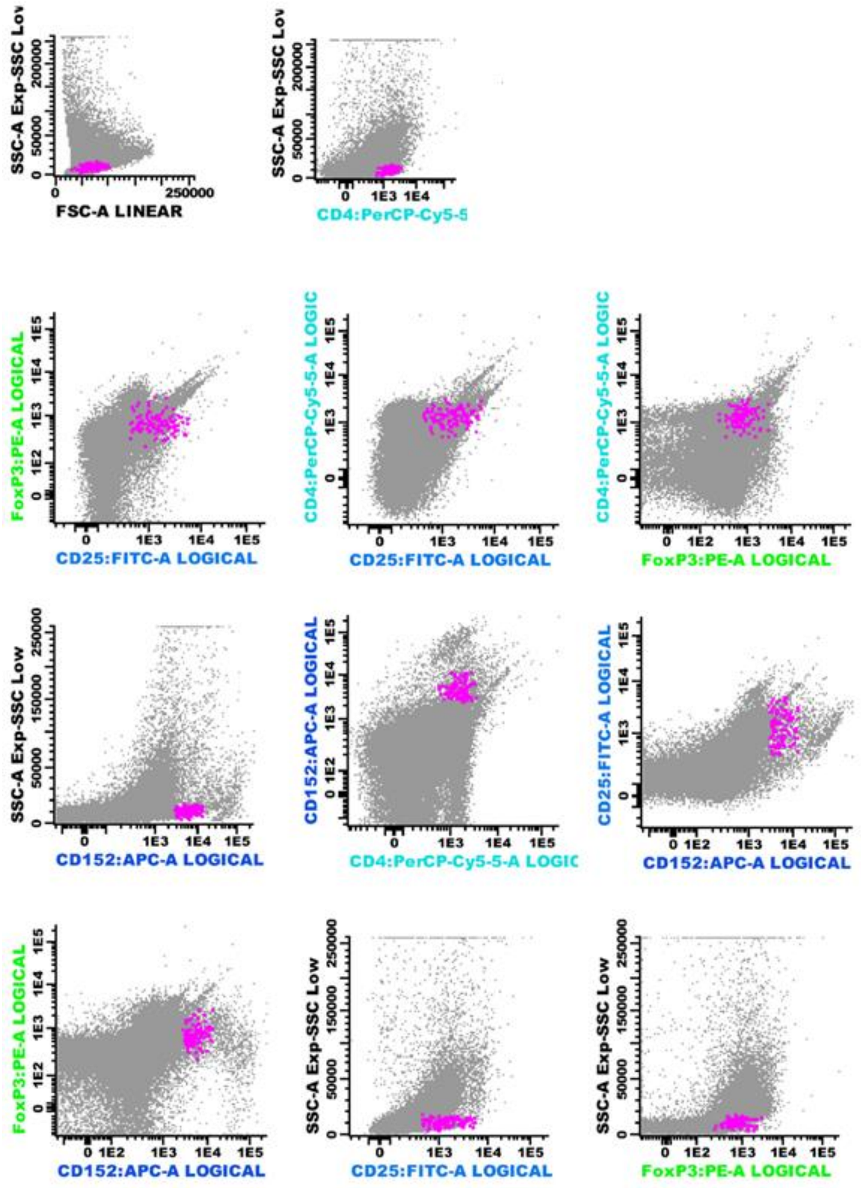

Supplementary Figure 1 C - Analysis of Treg cells by flow cytometry in total bone marrow aspirate: CD3+CD4+CD25highFOXP3+CTLA4(CD152)+ in a multiple myeloma case.
